# On order and disorder during the COVID‐19 pandemic

**DOI:** 10.1111/bjso.12398

**Published:** 2020-07-01

**Authors:** Stephen Reicher, Clifford Stott

**Affiliations:** ^1^ School of Psychology and Neuroscience University of St. Andrews UK; ^2^ School of Psychology University of Keele UK

**Keywords:** COVID‐19, social order, social disorder, shared social identity, leadership, procedural justice, policing

## Abstract

In this paper, we analyse the conditions under which the COVID‐19 pandemic will lead either to social order (adherence to measures put in place by authorities to control the pandemic) or to social disorder (resistance to such measures and the emergence of open conflict). Using examples from different countries (principally the United Kingdom, the United States, and France), we first isolate three factors which determine whether people accept or reject control measures. These are the historical context of state‐public relations, the nature of leadership during the pandemic and procedural justice in the development and operation of these measures. Second, we analyse the way the crisis is policed and how forms of policing determine whether dissent will escalate into open conflict. We conclude by considering the prospects for order/disorder as the pandemic unfolds.

## Background

What are the effects of hard times on human behaviour? There are two diametrically opposed answers to this question.

On the one hand, a large and growing literature on responses to crises, emergencies, and disasters shows how – in contrast to the traditional image of panic – people characteristically act in a controlled manner, they help and support each other, even at considerable risk to themselves, and this mutual aid extends even to strangers (Drury, 2018). Underlying this is a sense of shared identity which arises out of a common fate and which results in empathy and solidarity for one’s fellows. In short, hard times can create social cohesion and invoke widespread compassion (Drury & Alfadhli, 2019).

On the other hand, the historical record shows that some of the most extreme forms of social division and human hatred arise out of the psychology of hard times. Sometimes, this division is within the population as people become fearful and vent their fury on those identified as scapegoats (Staub, 1989). This is particularly true of pandemics and plagues. During the Black Death, for instance, some 2,000 Jews were burnt in Strasbourg on St. Valentine’s Day 1349 (Cohn, 2007). However, in this paper we focus on a different form of division – that between authorities and their public. Tensions, conflicts, and widespread rioting can often occur as limits on everyday activity are imposed as infection takes hold (Slack, 1985). For instance, Snowden, (1995) documents how, during the 1910 cholera epidemic in Italy, restrictions on burials led to attacks on health workers and the police.

In general terms, then, we need to understand what determines whether a pandemic leads to greater social cohesion or greater social conflict? The question is equally pertinent when it comes to the current pandemic. We have seen high levels of adherence to lockdown measures and a remarkable flowering of mutual aid groups – in Britain alone more than 4,000 groups involving some 3 million people (Butler, 2020). We have also seen discord and overt conflict between the public and authorities – in France, Italy, Brazil, and the United States, for example. So what determines why and when cohesion gives way to conflict, social order to social disorder?

## The origins of order

Let us turn first to the question of ‘order’. More specifically, we focus here on whether the public accept without dissent the things that governments demand of them. In the context of COVID‐19, why do people tolerate restrictions on their everyday lives, such as lockdown? The question of why people obey authorities is one of the most famous in the history of psychology. It is exemplified by the popularity and notoriety of Milgram’s Yale Obedience studies (Milgram, 1974). Yet, Milgram’s work is more influential as a demonstration of how far some people will go in obeying an authority figure than as an explanation of this phenomenon. Indeed, Milgram’s ‘agentic state’ account of obedience is little more than tautology – suggesting that we obey authority because we enter a state where we focus uniquely on obeying authority and hence automatically obey orders.

Our recent re‐analyses suggest a very different story. Obedient participants, we argue, should be understood as ‘engaged followers’ (Reicher & Haslam, 2019; Reicher *et al*., 2012). Far from being passive in the presence of an authority, they make an active ideological choice to work with the experimenter to advance the scientific enterprise – something they see as a noble cause. Moreover, we suggest this does not happen by accident. It is a result of active ‘identity leadership’ by the experimenter (Haslam *et al*., 2019) who actively seeks to bring about such shared identification between himself and the participant in the cause of science.

This conclusion chimes with that from the work of Tyler and colleagues on why people obey the law (Tyler, 1990) – the so‐called ‘group engagement’ model (Tyler & Blader, 2003). They show that adherence depends upon whether people see themselves and authorities – particularly the police – as part of a common in‐group. They too focus on the antecedents of such shared identity, but the emphasis is less on leadership than on procedural justice in the interactions between authorities and their publics. It is by giving people voice, by treating them fairly and with respect, that a sense of being part of a common group is created. This in turn legitimates laws and regulations and increases adherence to them (Bradford, Murphy & Jackson, 2014).

More recent research suggests that the potency of procedural justice lies not simply in creating an in‐group relationship between authorities and citizens but in establishing the authorities as prototypical in‐group members acting for and serving the interests of the group (Radburn & Stott, 2019; Radburn, Stott, Bradford & Robinson, 2018; Stott *et al*., 2012).

This work puts flesh on John Turner’s suggestion that the procedural justice framework ‘points to a whole range of other factors relevant to identification with authorities and acceptance of their control as an ingroup norm (e.g. the ideology and goals of group members, the social comparative context, their history of success or failure for the group, the degree to which the authorities are perceived as more or less prototypical of the relevant identity)’ (2005, p. 11). Echoing Turner, we suggest that, in addition to leadership and procedural justice, historical and structural context is a third antecedent of shared in‐group identity and hence of adherence to authority.

Let us now briefly consider the significance of these three factors in the COVID‐19 pandemic. We use, as an example, the United Kingdom – where levels of adherence to restrictions have been consistently high. Clearly, given that we are still in the midst of the pandemic and do not yet have the luxury of prolonged reflection or comprehensive data, our argument is more illustrative than definitive. But still, it is worth considering whether we can make sense of what we know thus far.

First, regarding historical context, Britain has a long tradition of treating state intervention as progressive and in the interests of the general population – which even if not a consistent reality, serves as a potent national myth (Rollings, 1996). Politically, this is expressed in the idea that policing is not an imposition from the centre but rooted in local consent (Channing, 2015). Economically, it is encapsulated in the idea of welfarism (Page & Silburn, 1999). Institutionally, it is exemplified by the NHS, invoked as a centrepiece in Danny Boyle’s pageant of Britishness for the opening ceremony of the 2012 London Olympics and currently occupying an almost deified status. Additionally, there are also powerful historical myths of state and society joining as one in times of national crisis as exemplified in the notion of a ‘Blitz spirit’ and of the King and Queen remaining in London to share the plight of the people during WW2 (Calder, 1992).

These historical myths are potent sources for the ways contemporary social realities are broadly understood, and social relations are defined in the present. But they are not so much determining cultural presences as rhetorical resources that can be drawn upon – and indeed need to be actively invoked – if they are to influence our understanding of COVID‐19, our relationship to authority during the pandemic and our responses to their policies. This takes us to the second antecedent of shared identity: leadership.

There are already many controversies about the leadership of the UK Government during this crisis and no doubt they will grow in time: did they tarry too long in declaring lockdown and have they shown sufficient diligence in developing testing and in securing protective equipment for frontline workers? Nonetheless, Government ministers and Government information services have, increasingly over time, framed the crisis in inclusive national terms – a ‘we’ thing that incorporates government and people in common cause^1^Note, this was written in early May before the ‘Cummings’ affair – in which a top government advisor was seen to break lockdown rules without repercussions – led to a strong sense of alienation from the Government.. And indeed the Queen, in her address to the nation on 5th April, invoked the myth of a ‘Blitz spirit’ to ground her calls for national solidarity, notably by finishing with a reference to Vera Lynn’s most famous of all WW2 songs: ‘we will meet again’.

But the rhetoric of ‘togetherness’ is of little use and may indeed be undermined when it is not matched by practices which make it possible for us to come together. Otherwise, the words will be seen as hypocrisy and will only serve to undermine a sense of equity and trust in authority. At this point, we are back in the territory of procedural justice – the third antecedent of identification with authority – albeit with a critical twist. That is, it is important that fairness is not just a matter of rhetoric and the process by which regulations are introduced but that it extends to the ways in which those regulations impact on people in practice (Armaline, Sanchez & Correia, 2014; Epp, Maynard‐Moody & Haider‐Markel, 2014).

Once again, there is already criticism of Government support to enable everyone to observe lockdown as too little and too late. And again, there will be more. There is little doubt that the poor and precarious are less able to stay at home, even if they are as motivated as the rich and secure to do so (Atchison *et al*., 2020). The evidence is clear that the poor (and BAME people) are getting infected and dying at far higher rates than the affluent (Pidd, Barr & Mohdin, 2020). But first, the United Kingdom does have a range of welfare provisions including free universal health care. Second, even if insufficient, nonetheless unprecedented financial measures have been implemented to help people to stay off work during the pandemic: a boost in welfare payments, a state guarantee to replace 80% of lost wages and of lost income for the self‐employed. This has been high enough to secure high levels of trust in Government and support for lockdown even amongst those suffering from it (Duffy & Allington, 2020). Enough, for now at least (as we write at the start of May), to secure the social order.

## The roots of disorder

Let us turn next to the question of ‘disorder’ and more specifically to protests against government social distancing measures and to anti‐authority rioting in the context of the pandemic. If a facilitative historical context, inclusive leadership, and the legitimacy of authority (cf. Bottoms & Tankebe, 2017) are critical to creating shared identity and maintaining social order, what happens when these factors are absent? Let us answer that question primarily by reference to the USA where (unlike in the United Kingdom) there have been multiple protests against lockdown – but additionally by addressing the unrest that has occurred in various other countries such as Italy, Brazil, and France. The caveats we expressed concerning our analysis of the UK case obviously apply here as well.

As before, we shall start with the historical relationship between state and people. Gary Wills starts his history of this relationship in the United States with a famous quotation: ‘*Henry David Thoreau put in extreme form what many Americans want to believe: “I heartily accept the motto: ‘the government is best which governs least*’”’ (2002, p.15). The country, after all, was born in a revolution against what was seen as an alien and tyrannical state, and this attitude of suspicion remained attached to any state power. To this day, the right to bear arms (as articulated in the 2nd amendment to the constitution) is seen by many as a necessary counterbalance to such tyranny. In this context, any and all state intervention has the potential to be rejected as unacceptable and anti‐American.

Once again, however, these beliefs do not exert a spontaneous influence. They have to be invoked, applied to the current context, and used to mobilize people against anti‐pandemic measures such as lockdown. Anti‐state leadership is critical and, remarkably, it was provided by the right‐wing populist Head of State, US President Donald Trump. He directly referenced the anti‐state principles on which ‘this country’ was built in order to criticize anti‐pandemic measures. He praised protestors against lockdown as ‘responsible’, and he tweeted ‘Liberate Minnesota!’ followed by ‘Liberate Michigan’ and then ‘Liberate Virginia and save your great 2nd Amendment. It is under siege!’ (Embury‐Dennis, 2020).

Finally, the huge structural inequalities in the United States, the fact that less than half of poorer workers get any sick pay, that 28.6 million people have no health cover, and that 40% of Americans could not find $400 to cover an emergency (Vesoulis, 2020), combined with the fact 26 million Americans became unemployed in March and April 2020 (Santhanam, 2020), have led to great hardship in the period of lockdown. This creates a context in which a sizeable pool of people is amenable to being mobilized by anti‐lockdown leadership. In the poor South, for instance, 38% of people support Trump on the pandemic over their own State governors (Santhanam, 2020). While, as yet, there are no systematic analyses of those who are protesting, anecdotal reports – along with scrutiny of the chants and the placards displayed in protests – suggest a combination of ‘liberty’ and ‘hardship’ concerns – a leadership and organization rooted in traditional anti‐state conservatism gaining some traction amongst those alienated from the authorities by economic suffering. As Stocpol puts it: ‘we’ve got a… combination of top‐down influence from high‐dollar organizations and some genuine energy at the grassroots level’ (cited in Illing, 2020).

In suggesting that all three factors – anti‐state historical context, anti‐state leadership, and inequity – contribute to the emergence of disorder, we do not suggest they are all equivalent or all necessary for protest to emerge. Thus, events in America show how history and leadership can facilitate the emergence of an understanding that pandemic measures are unfair and unjust – an illegitimate imposition by an alien authority that is acting against us rather than for and with us. However, events elsewhere – we shall focus particularly on France – show that such an understanding can arise directly from government measures that are experienced as inequitable and the ways that those who then challenge these measures are treated by the police.

## Policing and the proximal dynamics of conflict

The lockdown in France took effect on 17 March 2020. This was in the context of the long‐standing ‘Yellow Vest’ movement. As Jetten *et al.* (2020) show, a sense of popular alienation from a state out of touch with the sufferings of ordinary people intertwined with the notoriously aggressive heavy handedness of French policing to drive the movement towards violent protest and sustain these protests across time and location.

These features were then reproduced within the lockdown itself. As elsewhere, the lockdown exacerbated inequalities of class and ‘race’, making it particularly difficult for poor ethnic minority members to adhere to the regulations and stay at home (Willsher & Harrap, 2020). The state responded with heavy‐handed repression. Anyone leaving their home has been required to carry a time‐stamped certificate that has to be produced on demand. Infringement carries penalties of heavy fines or even up to six‐year imprisonment for the crime of ‘endangerment’. By 1st April, just sixteen days after the control measures were introduced, the French police had already carried out 5.8 million controls and issued 359,000 fines (Statista, 2020) compared to less than 10,000 across the one month following the imposition of control measures in the United Kingdom. (NPCC, 2020).

Just one week into lockdown, French media began to report that the police were experiencing difficulties in enforcing such repressive measures especially in the poorer and ethnically mixed Paris suburbs (Keiger, 2020), themselves historically areas of anti‐police sentiment and rioting (Body‐Gendrot, 2017). Then, early in the morning of 19th April, in the Paris suburb of Villeneuve‐la‐Garenne, a local man of Arab decent riding a motorcycle was injured when he collided with the open door of an unmarked police car. The incident was immediately interpreted by many as just another example of police brutality and rioting developed across the next four days, spreading into four suburbs across Paris and with additional incidents reported across France (Willsher, 2020).

What we see here is a classic pattern of crowd violence. A background of structural inequalities leading to alienation from authority, a pattern of antagonistic interactions with the police such that they become the concrete face of the ‘other’, a specific incident which is understood to exemplify the illegitimacy of the police, and then a spread of violence to other sites where locals share such a view of the police (Ball *et al*, 2019; Drury *et al*, 2020; Reicher & Stott, 2011, 2020; Stott *et al*, 2018).

The critical point is to understand how the distal determinants of conflict (which lie in the interaction of general structural inequalities and specific policies such as lockdown) interact with the proximal determinants (which lie in indiscriminate and repressive forms of policing, and in the spiral of negative interactions with particular sectors of the public that are initiated by such repression). It is only by developing such an understanding of how disorder occurs that we can learn how to preserve public order.

## Conclusion

We began this paper by asking what determines whether a pandemic brings people together with authorities so as to maintain order or else pushes them apart and creates disorder. Our answer has been at two levels. On the one hand, we must look at the combination of historical context and contemporary leadership which determine whether people will mobilize around perceived inequities to challenge authority and disregard the regulations they seek to impose. On the other hand, we have pointed to the specific role of policing – specifically repressive policing – in escalating dissent into open violence. Both are key domains for further investigation.

Nonetheless, even if the details of our analysis are necessarily provisional at this stage of events, it is clear that disorder is a complex phenomenon which requires a combination of structural, political, and interactional factors to occur. Consequently, lack of disorder might tell us that not *all* is bad, but it does not tell us that all is well either. Nor does it allow us to be complacent. That is certainly true of the situation in the United Kingdom.

As we have indicated, there are major structural inequalities which are reflected in the disproportionate death rates of poor and BAME people. Equally the leadership and the political response to the pandemic may have done just enough to preserve cohesion, but there are more and more voices expressing concerns about the inefficiencies and inequities of current Government policies – and these may grow as the situation develops. Finally, the policing response may have largely concentrated on the three Es of Engaging, Explaining, and Encouraging the public to adhere to government measures (such as lockdown) before reverting to the fourth E – enforcement (NPCC, 2020a). But the pattern has not been consistent across the country.

All in all, we cannot rest on our laurels. Unless we are clearly seen to do more in the longer term to make a priority of addressing the structural inequalities; unless more steps are taken to make it possible for those in precarious positions to cope with lockdown and other measures; unless the focus of the police and other agencies is more firmly focussed on enabling rather than enforcing coherence, then the precious and fragile social consensus that we have enjoyed thus far could always give way to social conflict.

## Author contributions

Stephen Reicher, PhD (Conceptualization; Writing – original draft) Clifford Stott (Conceptualization; Writing – review & editing).

## Conflicts of interest

All authors declare no conflict of interest.

## Data Availability

No data available.
